# Investigation on subcellular localization of Rice stripe virus in its vector small brown planthopper by electron microscopy

**DOI:** 10.1186/1743-422X-10-310

**Published:** 2013-10-18

**Authors:** Jinhua Deng, Shuo Li, Jian Hong, Yinghua Ji, Yijun Zhou

**Affiliations:** 1 Institute of Plant Protection, Jiangsu Academy of Agricultural Sciences; Jiangsu Technical Service Center of Diagnosis and Detection for Plant Virus Diseases, Nanjing 210014, People’s Republic of China; 2 Suzhou Academy of Agricultural Sciences, Suzhou 215100, People’s Republic of China; 3 Institute of Biotechnology, Zhejiang University, Hangzhou, 310058, People’s Republic of China

**Keywords:** Rice stripe virus, Small brown planthopper, Subcellular localization, Immuno-gold labeling, Transovarial transmission

## Abstract

**Background:**

Rice stripe virus (RSV), which is transmitted by small brown planthopper (*Laodelphax striatellus* Fallén, SBPH), has been reported to be epidemic and cause severe rice stripe disease in rice fields in many East Asian countries, including China. Investigation on viral localization in the vector is very important for elucidating transmission mechanisms of RSV by SBPH. In this study, transmission electron microscopy and immuno-gold labeling technique were used to investigate the subcellular localization of the ribonucleoproteins (RNPs) of RSV in the digestive tract, muscles, ovary and testes of SBPH.

**Results:**

A lot of amorphous RSV inclusion bodies with high electron density were observed in the cytoplasmic matrix and vacuoles of follicular cells of ovarioles in viruliferous SBPH, which were very similar to viral inclusions formed in rice cells. After magnified, it was found that sand-like or parallel filamentary structures were constructed inside the electron-dense inclusions. A large numbers of RSV RNPs distributed diffusely throughout the eggshell surface and interior of ovum, midgut lumen and epithelial cells, while the amount of the virus in muscles was far less than that in the ovary and midgut tissues. Besides RSV, numerous endogenous microorganisms were also observed in SBPH body, including yeast-like endosymbiotes (YLES), endosymbiotic bacteria and insect virus.

**Conclusions:**

According to the results of the virus localization, a potential mechanism of RSV transovarial transmission was proposed that RSV might replicate and accumulate initially in the inclusions of follicular cells, then exploit the pathway of the nutrition transportation to pass through the eggshell and spread into the oocytes along with the nutrition. Moreover, RSV might exploit muscles for its spread in vector body with a lower efficiency.

## Background

Rice stripe virus (RSV), the type member of the genus *Tenuivirus*, is currently present in subtropical and temperate regions in East Asian, and has been reported to cause severe losses in rice fields in China in last decades [1]. RSV is transmitted mainly by its vector small brown planthopper (*Laodelphax striatellus* Fallén, SBPH) in a persistent, circulative-propagative manner [2]. Female and male adults, nymphs all can transmit the virus, while SBPH nymphs were reported as more efficient vectors than adults, and females as more efficient vectors than males for RSV transmission [2]. After invading into SBPH, RSV can escape from midgut, salivary gland and ovary barriers and propagate in the body [3,4]. It has been confirmed that the ribonucleoproteins (RNPs) of RSV exist in follicular cells of the ovarioles and can be transmitted from female adults to their progeny via eggs [3]. Transovarial (vertical) transmission is an important characterization of RSV, which also increased difficulty of disease control. The epidemic and outbreak of rice stripe disease have close relationship with the outbreak of viruliferous populations of SBPH. Even at a lower density, viruliferous vectors could lead to significant yield losses by virus infection [5]. Moreover, latest research showed that SBPH could also transmit rice stripe disease to overseas rice fields through long-distance migration in East Asian countries [6]. Mass overseas migration of SBPH and a subsequent outbreak of rice stripe disease were reported to have occurred in western Japan in 2008 and western Korea in 2009 [7,8]. Therefore, it is crucial for disease control to research the mechanisms how RSV is transmitted specifically by SBPH.

Investigation on subcellular localization of virus in the vector is very important for understanding its transmission mechanisms. At present, the research on localization of RSV in SBPH has made certain progress. A preliminary study on the distribution situation of RSV in vector tissues and organs was conducted using conventional electron microscopy, which showed that the virus was observed in the principal salivary gland, midgut epithelial cells, follicular cells as well as fat body [3]. Wu et al. (2001) found nonstructural disease-specific protein (SP) of RSV located in the ovary, oocytes, intestinal cavity and epithelial cells of midgut in SBPH body via immuno-gold labeling technique [9]. Moreover, SP and N-terminal parts of NSvc2 (a putative membrane glycoprotein) were observed to co-localize in the midgut lumen and midgut epithelial cells of SBPH and form filamentous electron-opaque inclusion bodies (FEO), which suggested an interaction between SP and NSvc2 N-terminal [4]. In this paper, to further understand viral transmission mechanisms, we used transmission electron microscopy and immuno-gold labeling technique to investigate the meticulous subcellular localization of RSV in the midintestine cells, muscles, testes, ovary, follicular cells, ovum (or oocytes) and eggshell of SBPH.

## Results

### Ultrastructure observation through direct electron microscopy

**Figure 1:**
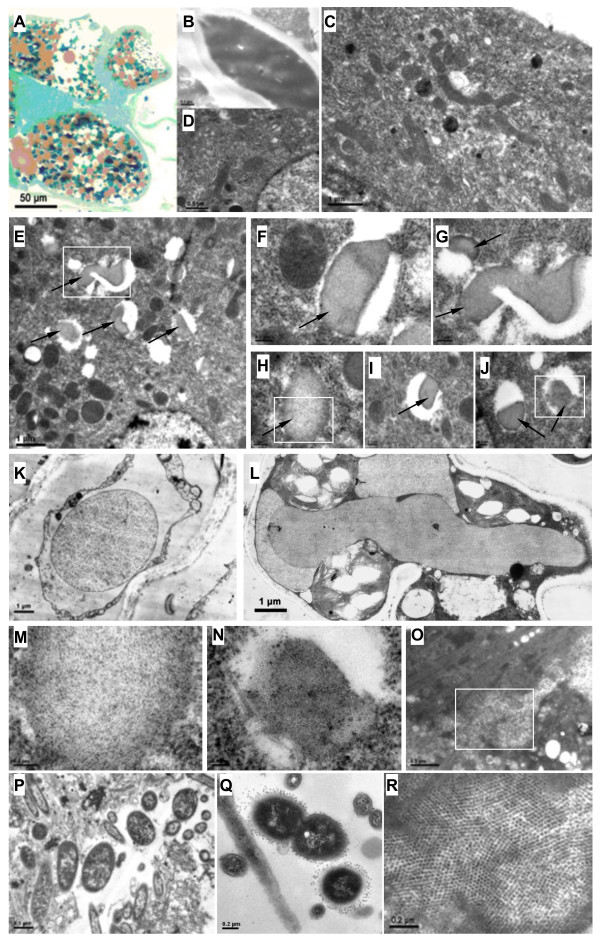
**Transmission electron micrographs showing RSV inclusion bodies and endogenous microorganisms in SBPH. A**: Light micrographs showing the transverse section of ovum with methylene blue staining. **B**: The magnified yeast-like endosymbiotes (YLES). **C**: The cytoplasmic matrix of follicular cells in the exuberant secretion phase. **D**: The follicular cells without inclusions in non-viruliferous SBPH. **E**-**J**, **M**, **N**: The amorphous RSV inclusion bodies in follicular cells of viruliferous SBPH. RSV inclusions were indicated by arrows. Panel G is an enlargement of the boxed area of panel E; Panel M is an enlargement of the boxed area of panel H to show parallel filamentary structures inside the inclusions; Panel N is an enlargement of the boxed area of panel J to show sand-like structures inside the inclusions. **K**, **L**: The RSV inclusions in rice cells. Panel K and L is cited from Hong et al. [10]. **O**, **R**: The crystal-like spherical virions in midgut cells of SBPH. Panel R is an enlargement of the boxed area of panel O. **P**, **Q**: Various endosymbiotic bacteria in the insect midgut. Scale bars: 50 μm **(A)**, 1 μm **(C, E, K, L)**, 0.5 μm **(B, D, O, P)**, 0.2 μm **(F, G, H, I, J, Q, R)** and 0.1 μm **(M, N)**.

Firstly, the ellipticum eggs of SBPH were observed by optical microscope. A large numbers of brown globular fat bodies and aquamarine yeast-like endosymbiotes (YLES) or their mycetocytes were observed in the transverse section of ovum via methylene blue staining (Figure 1A). After magnified by an electron microscope, majority YLES exhibited long fusiform (Figure 1B). In the ovarioles, there were a lot of follicular cells around oocytes, which transported nutrition into oocytes for development of ovum. Therefore, abundance rough endoplasmic reticulum (RER), mitochondria and Golgi apparatus existed in the cytoplasmic matrix of follicular cells in the exuberant secretion phase (Figure 1C). Besides aforementioned organelles, pleomorphic cytoplasmic inclusions with high electron density were also observed in the cytoplasmic matrix and vacuoles of follicular cells in viruliferous SBPH (Figure 1E), and no electron-dense inclusions were observed in follicular cells of non-viruliferous insect (Figure 1D). Shape of inclusion bodies was amorphous, including round, oval, lump, short-rod and irregular shape, etc. (Figure 1F, G, H, I, J). Formation of amorphous RSV inclusion bodies in rice cells were previously reported [10], which was very similar to these viral inclusions in SBPH cells (Figure 1K, L). After amplified and carefully observed, it was found that sand-like or parallel filamentary structures were constructed inside the electron-dense inclusions (Figure 1M, N), and the electron-dense inclusions might be considered as aggregates of viral proteins and insect components. Moreover, many endosymbiotic bacteria were observed in midgut lumen and midgut epithelial cells of SBPH, and some globular bacteria were attached closely by 16–18 nm spherical particles around bacterial capsules (Figure 1P, Q). These particles associated with bacteria might be bacteriophages. A large numbers of isometric spherical particles (approximately 25–29 nm in diameter) arranged together showing crystal-like form in midgut cells (Figure 1O, R), and these particles might be parasitic virions in the digestive system of SBPH.

### Subcellular localization of RSV by immuno-gold electron microscopy

**Figure 2:**
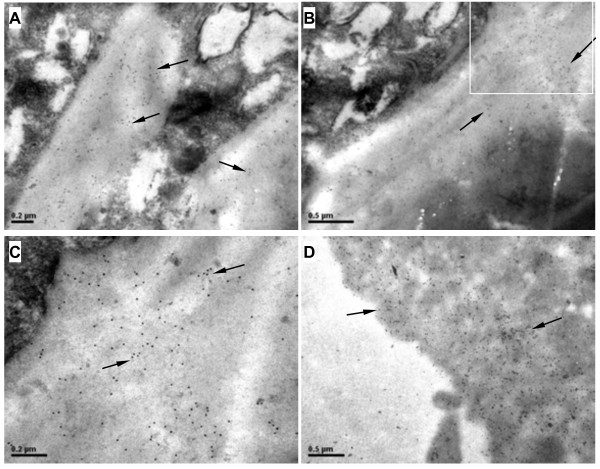
**Immuno-gold electron micrographs showing the subcellular localization of RSV RNP particles in viruliferous SBPH ovum. A**, **B**, **C**: The colloidal gold nanoparticles (RSV RNPs) distribute diffusely throughout the eggshell surface. Panel C is an enlargement of the boxed area of panel B. **D**: RSV RNPs in the interior of ovum. The virus RNPs are indicated by arrows. Scale bars: 0.2 μm **(A, C)** and 0.5 μm **(B, D)**.

**Figure 3:**
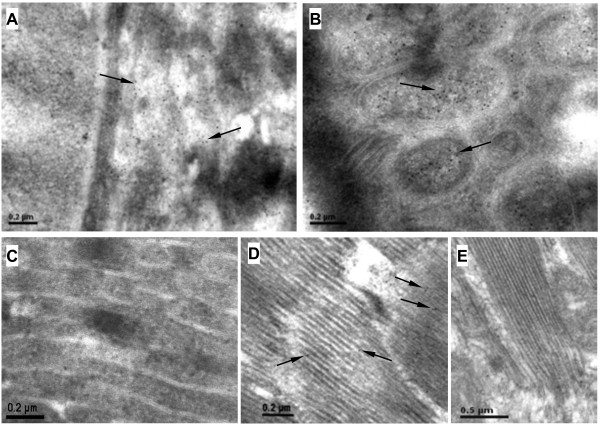
**Immuno-gold electron micrographs showing the subcellular localization of RSV RNPs in the midgut and muscles of viruliferous insect. A**: RSV RNP particles in the midgut lumen of SBPH. **B**: The virus invades into the insect midgut epithelial cells. **C**: The midgut epithelial cells of non-viruliferous SBPH as negative control. **D**: A few RNPs in the muscle tissues of RSV-infected SBPH. **E**: No RSV RNPs exist in muscles of non-viruliferous insects. RSV RNPs are indicated by arrows. Scale bars: 0.2 μm **(A, B, C, D)** and 0.5 μm **(E)**.

Immuno-gold labeling was applied to investigate subcellular localization of RSV RNP particles in viruliferous SBPH. When we examined material scraped from ovary by transmission electron microscopy, we found very large numbers of colloidal gold nanoparticles distributed diffusely throughout the eggshell surface (Figure 2A, B, C) and the interior regions of ovum (Figure 2D). RNP particles of the virus could be easily distinguished from other particles on the basis of shape and electron density (Figure 2C). Sections from intestinal tissue were examined, and the presence of RSV RNPs in the midgut lumen and epithelial columnar cells was observed (Figure 3A, B), while the midgut epithelial cells without the virus RNPs from non-viruliferous insect were also examined as negative control (Figure 3C). As shown in Figure 3D, a few of RSV RNPs were localized in the fibrillar muscle tissues of viruliferous SBPH, and the amount of the virus in muscles was far less than that in the ovary and midgut epithelial cells. No RNPs were found in muscles of non-viruliferous insect (Figure 3E). Furthermore, colloidal gold particles were scarcely observed in the testes and sperm of viruliferous SBPH (data was not shown).

## Discussion

In this paper, transmission electron microscopy and immuno-gold labeling were used to characterize viral inclusions and subcellular localization in RSV-infected SBPH, which facilitates to further understand transmission mechanisms of RSV. In the experiment, we observed no the particles of RSV RNPs in viral inclusions or insect cells via direct electron microscopy, which is considered reasonable. The RNPs of RSV have a thin filamentous shape (3–10 nm in diameter), with lengths proportional to the sizes of the RNAs they contain [11]. The filamentous particles may appear to be spiral-shaped, branched or circular [11]. Therefore, in the ultrathin sections of RSV-infected tissues, the particles of RSV RNPs are very difficult (almost impossible) to distinguish from host subcellular components.

Viral inclusions, known as viral factories, are confirmed as the replication and assembly sites of virus [12-14], which are also named viroplasms in the reoviruses. We found RSV inclusion bodies in SBPH cells were extremely similar with viral inclusions in rice plants. A large number of RSV amorphous inclusions aggregated in the cytoplasm of follicular cells of ovarioles, which indicates that the replication and assembly of RSV are more intensive in the follicular cells than in other tissues of viruliferous SBPH. In the ovarioles of SBPH, the monolayer follicular cells surround the developing oocytes and transport nutrition into oocytes [15]. Stronger accumulation of the virus in the follicular cells suggests that RSV might spread into the ovum along with the nutrition when the follicular cells transport nutrition to develop ovum, and the initial virus in the oocytes might derive from the follicular cells. Wu et al. (2012) also showed that there were numerous RSV RNPs aggregation in the follicular cells, germarium, and nutritive cords of the ovary with the immunofluorescence microscopy, and they considered that RSV entered in the oocytes together with the nutrition via the follicular cells and nutritive cords [15], which is basically consistent with our results. The latest research revealed the nutrition accompanied the virus in the process of transovarial transmission was the vitellogenin (unpublished). Moreover, uniform distribution of RSV RNP particles on the eggshell surface was observed, which suggestes the entering ovum sites of RSV might be disseminated and no regional specificity on the surface of ovum. As is known, there is the ovary barrier for the transovarial transmission of RSV in SBPH. According to the above mentioned, a potential mechanism of RSV transovarial transmission was proposed that the virus initially replicated and accumulated in the inclusion bodies of follicular cells, then exploited the pathway of the nutrition (vitellogenin) transportation to pass through the eggshell and spread into the oocytes along with the vitellogenin. Afterwards, RSV could continue to accumulate in SBPH eggs during the embryonic development.

About the viral components of RSV inclusions, it is still no systematic research. In general, constituents of viral inclusions contain viral multiple structural and non-structural proteins, as well as host cell components. For instance, the viroplasm of Rice dwarf virus (RDV) contains the non-structural proteins Pns6, Pns11, Pns12 and core proteins P1, P3, P5, P7 [12]. Jia et al. (2012) showed that non-structural protein P9-1 and major outer capsid protein P10, respectively, was one component of the viroplasm matrix of Southern rice black-streaked dwarf virus (SRBSDV) [16]. RSV is a single stranded RNA virus with four segmented genomes which contain 7 ORFs, and uses a negative and ambisense coding strategy for replication and infection in hosts [17]. Seven ORFs encode RNA-dependent RNA polymerase (RdRP), NS2 (a silencing suppressor with other unknown functions), NSvc2, NS3 (a suppressor of gene silencing), nucleocapsid protein (NCP), SP and movement protein NSvc4, respectively [18]. RSV RNP particles are composed of the genomic RNAs, NCP and RdRP [19], so RSV inclusions, as the replication and assembly sites of virus [12-14], contain definitely structural protein NCP and replicase RdRP. As previously reported, most electron-dense amorphous semi-electron-opaque inclusion bodies (dASO) of RSV contained only SP, while some dASO contained at least NS2, NSvc2 N-terminal, NS3, NCP as well as SP. The ring-like inclusions contained at least NSvc2 N-terminal, SP and NSvc4. Fibrillar amorphous semi-electron-opaque inclusion bodies (fASO) contained only SP, and the filamentous electron-opaque inclusion bodies (FEO) consisted of SP and NSvc2 N-terminal [4]. It is remarkable that dASO, fASO and ring-like inclusions were only found in RSV-infected rice tissues, whereas FEO was identified both in infected rice cells and in the midgut epithelial cells of SBPH. The amorphous RSV inclusions observed in this study are very similar to dASO described by Liang et al. [4], but different from FEO, which suggests that dASO might also exist in SBPH cells. Takahashi et al. (2003) demonstrated that RSV NS3 and NCP could aggregate in vivo and form inclusion bodies in *Spodoptera frugiperda* (Sf9) cells and in RSV-infected plant tissues [20]. The previously described RSV inclusions pattern suggests that the types of RSV inclusions might be distinguishing in its two hosts, and the styles are more abundant in rice plant than in SBPH. In addition, the constituents of viral inclusions might be different in the two hosts. In rice cells, all seven proteins are potentially involved in the formation of various inclusion bodies, and in SBPH, besides NS3, NCP, SP, NSvc2 and RdRP, whether NS2 and NSvc4 are components of viral inclusions remain to be further elucidated.

According to the subcellular localization results, there was the largest RSV accumulation in the ovary, followed by the midgut lumen and epithelial cells, and the least in the muscles. The midgut is a barrier where virus invades into insect vector through a transcytosis mechanism, and the ovary is also a barrier for the transovarial transmission of RSV. Therefore, numerous RSV RNPs aggregation in the two barriers is in line with the expectations. Since the sperm is not the carrier of RSV, the virus in insect offspring only derives from maternal parents when insects feed on uninfected plants. Massive accumulation of RSV in the ovary may be an adaptation for the interaction between RSV and SBPH, which facilitates its transovarial (vertical) transmission and propagation. A few viruses, such as RDV and Tomato spotted wilt virus (TSWV), could infect the vector visceral muscles to bypass conveniently the basal lamina barrier of the midgut [21,22]. Fewer presence of RSV in the muscles suggests that RSV might exploit vector’s muscles for its spread in SBPH body with a lower efficiency.

Numerous endogenous microorganisms were observed in SBPH body. The yeast-like endosymbiotes (YLES) were found in the fat bodies of ovum. Phylogenetic analysis has revealed that the YLES consists of fungal species from the ascomycetes and pyrenomycetes [23]. These YLES are transovarially inherited, and benefit their hosts by providing nutrition such as vitamins and sterols. The loss of YLES under high temperature can cause deleterious effect on the host insect [24]. The species of YLES have been identified through close examination of the combined EST library of SBPH, and 3061 unigenes that matched well with the 45 genera fungal species were retrieved [25]. Besides fungi, a variety of endosymbiotic bacteria were found in the digestive tract of SBPH, but bacteria species was not identified. At present, there is still no detailed research about the species of endosymbiotic bacteria in SBPH. In SBPH, *Wolbachia* infection is familiar and it was reported that the bacterium is distributed in the head, thorax, abdomen, salivary gland, guts, ovary and testis [26]. Zhang et al. (2010) showed the number of transcripts of *Wolbachia* in non-viruliferous SBPH was four-fold larger than that of viruliferous insect [25]. It is not clear whether this expression bias is associated with RSV infection. A 63 kDa chaperon GroEL protein, produced by bacterial endosymbionts, potentially plays a crucial role in virus transmission in aphid and whitefly by binding to virus particles and protecting them from rapid proteolytic degradation in gut and haemolymph [27-29]. So far, the interaction between GroEL and Tenuivirus has not been found. Li et al. (2011) used a virus overlay assay of protein blots to investigate the RSV-vector interactions, and did not find the binding between GroEL and RSV RNPs [30]. *Buchnera* GroEL is an important chaperon to protect virus particles in aphid and whitefly *B. tabaci*[27,31], so we searched *Buchnera groEL* sequence against the EST library of SBPH constructed by Zhang et al. [25], but no significant matches were retrieved. Moreover, no *groEL* genes from SBPH endosymbiotic bacteria have been cloned, except for *Wolbachia groEL*[32]. Whether the GroEL with a capability to protect RSV exists in SBPH remain to be further elucidated.

It is remarkable that a large numbers of crystal-like spherical virions were observed in SBPH midgut. According to their shape and diameter, we hypothesized that the parasitic virus might be Himetobi P virus (HiPV), an insect picorna-like virus. HiPV was originally isolated from SBPH when researchers attempted to purify RSV from its vector [33], which suggested a high concentration of HiPV in viruliferous (RSV) SBPH populations. A previous study reported there were high incidences of HiPV infection in several laboratory cultures of SBPH, while the virus was detected at a low incidence in SBPH populations in the field [34]. The insect samples used in this study were also from the cultures maintained in author’s laboratory for several years. HiPV infects obligately the midgut of host insects with an asymptomatic infection [35], while the midgut is precisely a vital barrier that RSV invades into SBPH. Furthermore, an interaction between RSV-NCP and one (VP1) of HiPV three major capsid proteins was also detected using a yeast two-hybrid screen by author’s laboratory (unpublished). As mentioned above, it was proposed that endosymbiotic HiPV in SBPH might affect potentially the replication and transmission of RSV through some unknown mechanisms. The hypothesis is worthy of further study.

## Materials and methods

### Preparation of virus and antibody

RSV-infected rice samples were collected from rice fields in Jiangsu Province, China. RSV RNP particles were purified from leaf material according to Toriyama [11]. Purified RNP particles were characterized by 12% SDS-PAGE, and immunized mouse to obtain the polyclonal antibody against RSV. The titre of antiserum was 1: 6400.

### Insect vector

SBPH populations used in this study were collected from Haian, Jiangsu Province, China (32.57˚ N, 120.45˚ E with an elevation of 5 m a.s.l.), and has been maintained as stock populations in the laboratory for nearly 6 years. High-viruliferous and non-viruliferous strains were screened and reared respectively in glass beakers, and the proportion of viruliferous SBPH was above 90% via dot immunobinding assay (DIBA) as described by Wang et al. [1]. Rice plants (cultivar Wuyujing No.3) as insect’s diet were grown in soil at 25°C with a photoperiod of 16 hrs/8 hrs (light/dark) in a growth incubator. After insects were introduced into a glass beaker which contained rice seedlings (2–3 cm high), the beaker was enclosed with a piece of nylon mesh. The planthoppers were transferred to fresh seedlings every 10–14 days to assure sufficient nutrition.

Viruliferous and non-viruliferous SBPH adults were used for anatomy. The insects were anesthetized with aether and immobilized with 2.5% glutaraldehyde solution. The heads, wings, legs and rear of thorax were removed, and then the alimentary canal, testis, ovary and ovum were dissected carefully with a sterile dissecting forceps.

### Transmission electron microscopy (TEM)

For electron microscopic analyses, tissue samples of SBPH were prepared as described by Marrie et al. [36], with some modifications. The materials were fixed in 2.5% (v/v) glutaraldehyde in 0.1 M phosphate buffer (pH6.8) for 24 h at 4°C, and postfixed in 1% osmium tetroxide in phosphate buffer. After washed five times for 30 min each in phosphate buffer, samples were dehydrated through a series of ethanol washes. After further dehydration in propylene oxide, the specimens were embedded in Spurr low-viscosity embedding resin, and sectioned into 1 μm or 70 nm sections with an Ultracut E ultramicrotome (Reichart-Jung) fitted with a 45˚ diamond knife. Sections (1 μm) were stained with 0.5% methylene blue on glass slides, and observed with optical microscope (Olympus, Japan). Ultrathin sections (70 nm) from the same embedding blocks were stained with uranyl acetate followed by lead citrate for 15 min respectively, and examined with a JEM-1200EX transmission electron microscope (JEOL, Japan) at an acceleration voltage of 60 kV.

### Immuno-gold labeling of ultrathin sections

Immuno-gold labeling study was performed as described by Liang et al. [4] and Daniel et al. [37], with some modifications. Tissue samples for labeling were fixed in 0.1 M phosphate buffer containing 4% (v/v) paraformaldehyde and 1% (v/v) glutaraldehyde for 3 h at 4°C. After washed five times in phosphate buffer, samples were dehydrated in a graded ethanol series at 4°C and embedded in Lowicryl K_4_M low temperature resin with infiltration for 1 week at -20°C before ultraviolet light (360 nm) irradiation polymerization for 72 h at -20°C and 24 h at room temperature. Thereafter, ultrathin sections (70 nm) were prepared with an Ultracut E ultramicrotome and collected with copper grids. The utrathin sections on copper grids were moistened in double-distilled water for 5 min, and blocked in blocking buffer (50 mM PBS containing 1% BSA, 0.02% PEG2000 and 0.1% NaN_3_) for 30 min. The sections were subsequently incubated with polyclonal antibody against RSV (1: 400 dilution) in blocking buffer followed by a colloidal gold-conjugated goat anti-mouse immunoglobulin G (Sigma-Aldrich) (secondary antibody, 1: 100 dilution) in blocking buffer for 1 h respectively. After washing three times for 10 min each with double-distilled water, poststaining and examination of sections were performed as described above for TEM. To investigate the specificity of immuno-labeling, a control experiment was conducted after omission of the primary incubation stage with RSV antiserum. Additional labeling study was also performed on non-viruliferous SBPH samples to provide a substrate control.

## Competing interests

The authors declare that they have no competing interests.

## Authors’ contributions

YZ and SL designed the study. JD and JH performed most experiments. SL, JH, JD, YJ and YZ analyzed the data. SL and YZ wrote and finalized the manuscript. All authors read and approved the final manuscript.
